# Ten Simple Rules for Creating a Good Data Management Plan

**DOI:** 10.1371/journal.pcbi.1004525

**Published:** 2015-10-22

**Authors:** William K. Michener

**Affiliations:** College of University Libraries & Learning Sciences, University of New Mexico, Albuquerque, New Mexico, United States of America; National Institutes of Health, UNITED STATES

## Introduction

**Fig 1:**
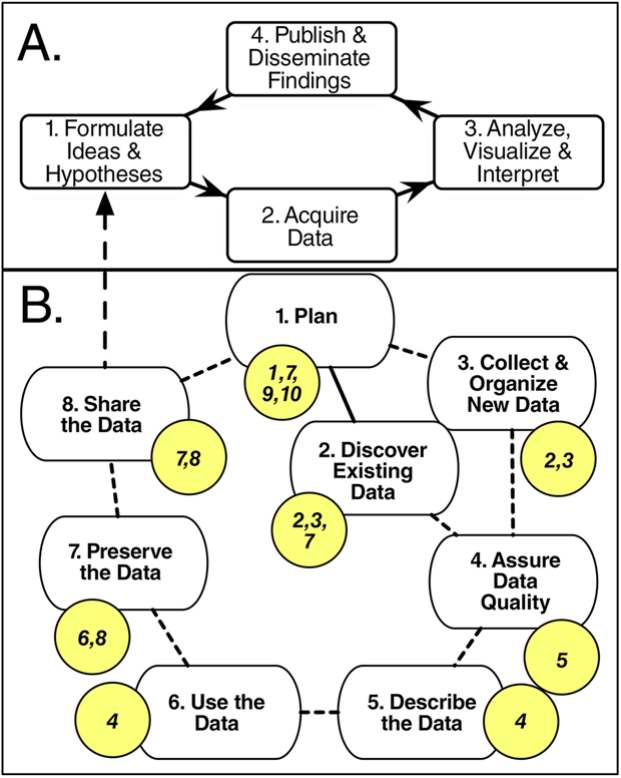
Relationship of the research life cycle (A) to the data life cycle (B); note: highlighted circles refer to the rules that are most closely linked to the steps of the data life cycle. As part of the research life cycle (A), many researchers (1) test ideas and hypotheses by (2) acquiring data that are (3) incorporated into various analyses and visualizations, leading to interpretations that are then (4) published in the literature and disseminated via other mechanisms (e.g., conference presentations, blogs, tweets), and that often lead back to (1) new ideas and hypotheses. During the data life cycle (B), researchers typically (1) develop a plan for how data will be managed during and after the project; (2) discover and acquire existing data and (3) collect and organize new data; (4) assure the quality of the data; (5) describe the data (i.e., ascribe metadata); (6) use the data in analyses, models, visualizations, etc.; and (7) preserve and (8) share the data with others (e.g., researchers, students, decision makers), possibly leading to new ideas and hypotheses.

Research papers and data products are key outcomes of the science enterprise. Governmental, nongovernmental, and private foundation sponsors of research are increasingly recognizing the value of research data. As a result, most funders now require that sufficiently detailed data management plans be submitted as part of a research proposal. A data management plan (DMP) is a document that describes how you will treat your data during a project and what happens with the data after the project ends. Such plans typically cover all or portions of the data life cycle—from data discovery, collection, and organization (e.g., spreadsheets, databases), through quality assurance/quality control, documentation (e.g., data types, laboratory methods) and use of the data, to data preservation and sharing with others (e.g., data policies and dissemination approaches). Fig 1 illustrates the relationship between hypothetical research and data life cycles and highlights the links to the rules presented in this paper. The DMP undergoes peer review and is used in part to evaluate a project’s merit. Plans also document the data management activities associated with funded projects and may be revisited during performance reviews.

Earlier articles in the Ten Simple Rules series of *PLOS Computational Biology* provided guidance on getting grants [1], writing research papers [2], presenting research findings [3], and caring for scientific data [4]. Here, I present ten simple rules that can help guide the process of creating an effective plan for managing research data—the basis for the project’s findings, research papers, and data products. I focus on the principles and practices that will result in a DMP that can be easily understood by others and put to use by your research team. Moreover, following the ten simple rules will help ensure that your data are safe and sharable and that your project maximizes the funder’s return on investment.

## Rule 1: Determine the Research Sponsor Requirements

Research communities typically develop their own standard methods and approaches for managing and disseminating data. Likewise, research sponsors often have very specific DMP expectations. For instance, the Wellcome Trust, the Gordon and Betty Moore Foundation (GBMF), the United States National Institutes of Health (NIH), and the US National Science Foundation (NSF) all fund computational biology research but differ markedly in their DMP requirements. The GBMF, for instance, requires that potential grantees develop a comprehensive DMP in conjunction with their program officer that answers dozens of specific questions. In contrast, NIH requirements are much less detailed and primarily ask that potential grantees explain how data will be shared or provide reasons as to why the data cannot be shared. Furthermore, a single research sponsor (such as the NSF) may have different requirements that are established for individual divisions and programs within the organization. Note that plan requirements may not be labeled as such; for example, the National Institutes of Health guidelines focus largely on data sharing and are found in a document entitled “NIH Data Sharing Policy and Implementation Guidance” (http://grants.nih.gov/grants/policy/data_sharing/data_sharing_guidance.htm).

Significant time and effort can be saved by first understanding the requirements set forth by the organization to which you are submitting a proposal. Research sponsors normally provide DMP requirements in either the public request for proposals (RFP) or in an online grant proposal guide. The DMPTool (https://dmptool.org/) and DMPonline (https://dmponline.dcc.ac.uk/) websites are also extremely valuable resources that provide updated funding agency plan requirements (for the US and United Kingdom, respectively) in the form of templates that are usually accompanied with annotated advice for filling in the template. The DMPTool website also includes numerous example plans that have been published by DMPTool users. Such examples provide an indication of the depth and breadth of detail that are normally included in a plan and often lead to new ideas that can be incorporated in your plan.

Regardless of whether you have previously submitted proposals to a particular funding program, it is always important to check the latest RFP, as well as the research sponsor’s website, to verify whether requirements have recently changed and how. Furthermore, don’t hesitate to contact the responsible program officer(s) that are listed in a specific solicitation to discuss sponsor requirements or to address specific questions that arise as you are creating a DMP for your proposed project. Keep in mind that the principle objective should be to create a plan that will be useful for your project. Thus, good data management plans can and often do contain more information than is minimally required by the research sponsor. Note, though, that some sponsors constrain the length of DMPs (e.g., two-page limit); in such cases, a synopsis of your more comprehensive plan can be provided, and it may be permissible to include an appendix, supplementary file, or link.

## Rule 2: Identify the Data to Be Collected

Every component of the DMP depends upon knowing how much and what types of data will be collected. Data volume is clearly important, as it normally costs more in terms of infrastructure and personnel time to manage 10 terabytes of data than 10 megabytes. But, other characteristics of the data also affect costs as well as metadata, data quality assurance and preservation strategies, and even data policies. A good plan will include information that is sufficient to understand the nature of the data that is collected, including:

- **Types.** A good first step is to list the various types of data that you expect to collect or create. This may include text, spreadsheets, software and algorithms, models, images and movies, audio files, and patient records. Note that many research sponsors define data broadly to include physical collections, software and code, and curriculum materials.
- **Sources.** Data may come from direct human observation, laboratory and field instruments, experiments, simulations, and compilations of data from other studies. Reviewers and sponsors may be particularly interested in understanding if data are proprietary, are being compiled from other studies, pertain to human subjects, or are otherwise subject to restrictions in their use or redistribution.
- **Volume.** Both the total volume of data and the total number of files that are expected to be collected can affect all other data management activities.
- **Data and file formats.** Technology changes and formats that are acceptable today may soon be obsolete. Good choices include those formats that are nonproprietary, based upon open standards, and widely adopted and preferred by the scientific community (e.g., Comma Separated Values [CSV] over Excel [.xls, xlsx]). Data are more likely to be accessible for the long term if they are uncompressed, unencrypted, and stored using standard character encodings such as UTF-16.

The precise types, sources, volume, and formats of data may not be known beforehand, depending on the nature and uniqueness of the research. In such case, the solution is to iteratively update the plan (see Rule 9).

## Rule 3: Define How the Data Will Be Organized

Once there is an understanding of the volume and types of data to be collected, a next obvious step is to define how the data will be organized and managed. For many projects, a small number of data tables will be generated that can be effectively managed with commercial or open source spreadsheet programs like Excel and OpenOffice Calc. Larger data volumes and usage constraints may require the use of relational database management systems (RDBMS) for linked data tables like ORACLE or mySQL, or a Geographic Information System (GIS) for geospatial data layers like ArcGIS, GRASS, or QGIS.

The details about how the data will be organized and managed could fill many pages of text and, in fact, should be recorded as the project evolves. However, in drafting a DMP, it is most helpful to initially focus on the types and, possibly, names of the products that will be used. The software tools that are employed in a project should be amenable to the anticipated tasks. A spreadsheet program, for example, would be insufficient for a project in which terabytes of data are expected to be generated, and a sophisticated RDMBS may be overkill for a project in which only a few small data tables will be created. Furthermore, projects dependent upon a GIS or RDBMS may entail considerable software costs and design and programming effort that should be planned and budgeted for upfront (see Rules 9 and 10). Depending on sponsor requirements and space constraints, it may also be useful to specify conventions for file naming, persistent unique identifiers (e.g., Digital Object Identifiers [DOIs]), and versioning control (for both software and data products).

## Rule 4: Explain How the Data Will Be Documented

Rows and columns of numbers and characters have little to no meaning unless they are documented in some fashion. Metadata—the details about what, where, when, why, and how the data were collected, processed, and interpreted—provide the information that enables data and files to be discovered, used, and properly cited. Metadata include descriptions of how data and files are named, physically structured, and stored as well as details about the experiments, analytical methods, and research context. It is generally the case that the utility and longevity of data relate directly to how complete and comprehensive the metadata are. The amount of effort devoted to creating comprehensive metadata may vary substantially based on the complexity, types, and volume of data.

A sound documentation strategy can be based on three steps. First, identify the types of information that should be captured to enable a researcher like you to discover, access, interpret, use, and cite your data. Second, determine whether there is a community-based metadata schema or standard (i.e., preferred sets of metadata elements) that can be adopted. As examples, variations of the Dublin Core Metadata Initiative Abstract Model are used for many types of data and other resources, ISO (International Organization for Standardization) 19115 is used for geospatial data, ISA-Tab file format is used for experimental metadata, and Ecological Metadata Language (EML) is used for many types of environmental data. In many cases, a specific metadata content standard will be recommended by a target data repository, archive, or domain professional organization. Third, identify software tools that can be employed to create and manage metadata content (e.g., Metavist, Morpho). In lieu of existing tools, text files (e.g., readme.txt) that include the relevant metadata can be included as headers to the data files.

A best practice is to assign a responsible person to maintain an electronic lab notebook, in which all project details are maintained. The notebook should ideally be routinely reviewed and revised by another team member, as well as duplicated (see Rules 6 and 9). The metadata recorded in the notebook provide the basis for the metadata that will be associated with data products that are to be stored, reused, and shared.

## Rule 5: Describe How Data Quality Will Be Assured

Quality assurance and quality control (QA/QC) refer to the processes that are employed to measure, assess, and improve the quality of products (e.g., data, software, etc.). It may be necessary to follow specific QA/QC guidelines depending on the nature of a study and research sponsorship; such requirements, if they exist, are normally stated in the RFP. Regardless, it is good practice to describe the QA/QC measures that you plan to employ in your project. Such measures may encompass training activities, instrument calibration and verification tests, double-blind data entry, and statistical and visualization approaches to error detection. Simple graphical data exploration approaches (e.g., scatterplots, mapping) can be invaluable for detecting anomalies and errors.

## Rule 6: Present a Sound Data Storage and Preservation Strategy

A common mistake of inexperienced (and even many experienced) researchers is to assume that their personal computer and website will live forever. They fail to routinely duplicate their data during the course of the project and do not see the benefit of archiving data in a secure location for the long term. Inevitably, though, papers get lost, hard disks crash, URLs break, and tapes and other media degrade, with the result that the data become unavailable for use by both the originators and others. Thus, data storage and preservation are central to any good data management plan. Give careful consideration to three questions:

1. How long will the data be accessible?
2. How will data be stored and protected over the duration of the project?
3. How will data be preserved and made available for future use?

The answer to the first question depends on several factors. First, determine whether the research sponsor or your home institution have any specific requirements. Usually, all data do not need to be retained, and those that do need not be retained forever. Second, consider the intrinsic value of the data. Observations of phenomena that cannot be repeated (e.g., astronomical and environmental events) may need to be stored indefinitely. Data from easily repeatable experiments may only need to be stored for a short period. Simulations may only need to have the source code, initial conditions, and verification data stored. In addition to explaining how data will be selected for short-term storage and long-term preservation, remember to also highlight your plans for the accompanying metadata and related code and algorithms that will allow others to interpret and use the data (see Rule 4).

Develop a sound plan for storing and protecting data over the life of the project. A good approach is to store at least three copies in at least two geographically distributed locations (e.g., original location such as a desktop computer, an external hard drive, and one or more remote sites) and to adopt a regular schedule for duplicating the data (i.e., backup). Remote locations may include an offsite collaborator’s laboratory, an institutional repository (e.g., your departmental, university, or organization’s repository if located in a different building), or a commercial service, such as those offered by Amazon, Dropbox, Google, and Microsoft. The backup schedule should also include testing to ensure that stored data files can be retrieved.

Your best bet for being able to access the data 20 years beyond the life of the project will likely require a more robust solution (i.e., question 3 above). Seek advice from colleagues and librarians to identify an appropriate data repository for your research domain. Many disciplines maintain specific repositories such as GenBank for nucleotide sequence data and the Protein Data Bank for protein sequences. Likewise, many universities and organizations also host institutional repositories, and there are numerous general science data repositories such as Dryad (http://datadryad.org/), figshare (http://figshare.com/), and Zenodo (http://zenodo.org/). Alternatively, one can easily search for discipline-specific and general-use repositories via online catalogs such as http://www.re3data.org/ (i.e., REgistry of REsearch data REpositories) and http://www.biosharing.org (i.e., BioSharing). It is often considered good practice to deposit code in a host repository like GitHub that specializes in source code management as well as some types of data like large files and tabular data (see https://github.com/). Make note of any repository-specific policies (e.g., data privacy and security, requirements to submit associated code) and costs for data submission, curation, and backup that should be included in the DMP and the proposal budget.

## Rule 7: Define the Project’s Data Policies

Despite what may be a natural proclivity to avoid policy and legal matters, researchers cannot afford to do so when it comes to data. Research sponsors, institutions that host research, and scientists all have a role in and obligation for promoting responsible and ethical behavior. Consequently, many research sponsors require that DMPs include explicit policy statements about how data will be managed and shared. Such policies include:

- licensing or sharing arrangements that pertain to the use of preexisting materials;
- plans for retaining, licensing, sharing, and embargoing (i.e., limiting use by others for a period of time) data, code, and other materials; and
- legal and ethical restrictions on access and use of human subject and other sensitive data.

Unfortunately, policies and laws often appear or are, in fact, confusing or contradictory. Furthermore, policies that apply within a single organization or in a given country may not apply elsewhere. When in doubt, consult your institution’s office of sponsored research, the relevant Institutional Review Board, or the program officer(s) assigned to the program to which you are applying for support.

Despite these caveats, it is usually possible to develop a sound policy by following a few simple steps. First, if preexisting materials, such as data and code, are being used, identify and include a description of the relevant licensing and sharing arrangements in your DMP. Explain how third party software or libraries are used in the creation and release of new software. Note that proprietary and intellectual property rights (IPR) laws and export control regulations may limit the extent to which code and software can be shared.

Second, explain how and when the data and other research products will be made available. Be sure to explain any embargo periods or delays such as publication or patent reasons. A common practice is to make data broadly available at the time of publication, or in the case of graduate students, at the time the graduate degree is awarded. Whenever possible, apply standard rights waivers or licenses, such as those established by Open Data Commons (ODC) and Creative Commons (CC), that guide subsequent use of data and other intellectual products (see http://creativecommons.org/ and http://opendatacommons.org/licenses/pddl/summary/). The CC0 license and the ODC Public Domain Dedication and License, for example, promote unrestricted sharing and data use. Nonstandard licenses and waivers can be a significant barrier to reuse.

Third, explain how human subject and other sensitive data will be treated (e.g., see http://privacyruleandresearch.nih.gov/ for information pertaining to human health research regulations set forth in the US Health Insurance Portability and Accountability Act). Many research sponsors require that investigators engaged in human subject research approaches seek or receive prior approval from the appropriate Institutional Review Board before a grant proposal is submitted and, certainly, receive approval before the actual research is undertaken. Approvals may require that informed consent be granted, that data are anonymized, or that use is restricted in some fashion.

## Rule 8: Describe How the Data Will Be Disseminated

The best-laid preservation plans and data sharing policies do not necessarily mean that a project’s data will see the light of day. Reviewers and research sponsors will be reassured that this will not be the case if you have spelled out how and when the data products will be disseminated to others, especially people outside your research group. There are passive and active ways to disseminate data. Passive approaches include posting data on a project or personal website or mailing or emailing data upon request, although the latter can be problematic when dealing with large data and bandwidth constraints. More active, robust, and preferred approaches include: (1) publishing the data in an open repository or archive (see Rule 6); (2) submitting the data (or subsets thereof) as appendices or supplements to journal articles, such as is commonly done with the PLOS family of journals; and (3) publishing the data, metadata, and relevant code as a “data paper” [5]. Data papers can be published in various journals, including *Scientific Data* (from Nature Publishing Group), the *GeoScience Data Journal* (a Wiley publication on behalf of the Royal Meteorological Society), and *GigaScience* (a joint BioMed Central and Springer publication that supports big data from many biology and life science disciplines).

A good dissemination plan includes a few concise statements. State when, how, and what data products will be made available. Generally, making data available to the greatest extent and with the fewest possible restrictions at the time of publication or project completion is encouraged. The more proactive approaches described above are greatly preferred over mailing or emailing data and will likely save significant time and money in the long run, as the data curation and sharing will be supported by the appropriate journals and repositories or archives. Furthermore, many journals and repositories provide guidelines and mechanisms for how others can appropriately cite your data, including digital object identifiers, and recommended citation formats; this helps ensure that you receive credit for the data products you create. Keep in mind that the data will be more usable and interpretable by you and others if the data are disseminated using standard, nonproprietary approaches and if the data are accompanied by metadata and associated code that is used for data processing.

## Rule 9: Assign Roles and Responsibilities

A comprehensive DMP clearly articulates the roles and responsibilities of every named individual and organization associated with the project. Roles may include data collection, data entry, QA/QC, metadata creation and management, backup, data preparation and submission to an archive, and systems administration. Consider time allocations and levels of expertise needed by staff. For small to medium size projects, a single student or postdoctoral associate who is collecting and processing the data may easily assume most or all of the data management tasks. In contrast, large, multi-investigator projects may benefit from having a dedicated staff person(s) assigned to data management.

Treat your DMP as a living document and revisit it frequently (e.g., quarterly basis). Assign a project team member to revise the plan, reflecting any new changes in protocols and policies. It is good practice to track any changes in a revision history that lists the dates that any changes were made to the plan along with the details about those changes, including who made them.

Reviewers and sponsors may be especially interested in knowing how adherence to the data management plan will be assessed and demonstrated, as well as how, and by whom, data will be managed and made available after the project concludes. With respect to the latter, it is often sufficient to include a pointer to the policies and procedures that are followed by the repository where you plan to deposit your data. Be sure to note any contributions by nonproject staff, such as any repository, systems administration, backup, training, or high-performance computing support provided by your institution.

## Rule 10: Prepare a Realistic Budget

Creating, managing, publishing, and sharing high-quality data is as much a part of the 21st century research enterprise as is publishing the results. Data management is not new—rather, it is something that all researchers already do. Nonetheless, a common mistake in developing a DMP is forgetting to budget for the activities. Data management takes time and costs money in terms of software, hardware, and personnel. Review your plan and make sure that there are lines in the budget to support the people that manage the data (see Rule 9) as well as pay for the requisite hardware, software, and services. Check with the preferred data repository (see Rule 6) so that requisite fees and services are budgeted appropriately. As space allows, facilitate reviewers by pointing to specific lines or sections in the budget and budget justification pages. Experienced reviewers will be on the lookout for unfunded components, but they will also recognize that greater or lesser investments in data management depend upon the nature of the research and the types of data.

## Conclusion

A data management plan should provide you and others with an easy-to-follow road map that will guide and explain how data are treated throughout the life of the project and after the project is completed. The ten simple rules presented here are designed to aid you in writing a good plan that is logical and comprehensive, that will pass muster with reviewers and research sponsors, and that you can put into practice should your project be funded. A DMP provides a vehicle for conveying information to and setting expectations for your project team during both the proposal and project planning stages, as well as during project team meetings later, when the project is underway. That said, no plan is perfect. Plans do become better through use. The best plans are “living documents” that are periodically reviewed and revised as necessary according to needs and any changes in protocols (e.g., metadata, QA/QC, storage), policy, technology, and staff, as well as reused, in that the most successful parts of the plan are incorporated into subsequent projects. A public, machine-readable, and openly licensed DMP is much more likely to be incorporated into future projects and to have higher impact; such increased transparency in the research funding process (e.g., publication of proposals and DMPs) can assist researchers and sponsors in discovering data and potential collaborators, educating about data management, and monitoring policy compliance [6].
